# Epidemiological Insights into Maedi-Visna Virus in Algeria: First National Seroprevalence Survey and Risk Factor Profiling in Sheep Herds

**DOI:** 10.3390/ani15152166

**Published:** 2025-07-23

**Authors:** Takfarinas Idres, Nasir Adam Ibrahim, Ali Lamara, Sofiane Boudjellaba, Assia Derguini, Nosiba Sulaiman Basher, Soraya Temim, Mohammed Saad Aleissa, Yahia Chebloune

**Affiliations:** 1 Laboratory for Livestock Animal Production and Health Research, Rabie Bouchama National Veterinary School of Algiers, Issad ABBAS Street, BP 161 Oued Semar, Algiers 16059, Algeria; a.lamara@ensv.dz (A.L.); s.temim@ensv.dz (S.T.); 2 Biology Department, College of Science, Imam Mohammad Ibn Saud Islamic University (IMSIU), Riyadh 11623, Saudi Arabia; naabdalneim@imamu.edu.sa (N.A.I.); nsbasher@imamu.edu.sa (N.S.B.); msaleissa@imamu.edu.sa (M.S.A.); 3 Research Laboratory for Management of Local Animal Resources, Rabie Bouchama National Veterinary School of Algiers, Issad ABBAS Street, BP 161 Oued Semar, Algiers 16059, Algeria; s.boudjellaba@ensv.dz; 4 Microbial Ecology Laboratory, Faculté des Sciences de la Nature et de la Vie, Abderrahmane MIRA University, Bejaïa 06000, Algeria; assia.derguini@univ-bejaia.dz; 5 Pathogenesis and Lentivirus Vaccination Laboratory, Grenoble Alpes University, 570 rue de la Chimie, Saint Martin d’Hères, 38400 Grenoble, France; yahia.chebloune@univ-grenoble-alpe.fr

**Keywords:** maedi-visna virus, small ruminant lentiviruses, seroprevalence, Algeria, risk factors, progressive pneumonia

## Abstract

Maedi-visna virus (MVV), a small ruminant lentivirus causing chronic multisystemic disease in sheep, poses significant economic burdens due to reduced productivity and a lack of effective treatments. Despite its worldwide prevalence, epidemiological data from Algeria remain absent. This first national seroprevalence study aimed to elucidate MVV distribution, risk factors, and transmission dynamics in Algerian sheep herds. A cross-sectional survey of 1400 sheep across four regions (East, Center, West, South) was conducted, with sera analyzed via indirect ELISA (IDvet). Risk factors (geography, age, sex, breed, farming system) were evaluated using chi-square tests and Cramer’s V. Overall seroprevalence was 9.07% (95% CI: 7.57–10.57), with significant variation by sex (females: 20.44% vs. males: 3.68%; *p* < 0.05), age (1–5 years: 6.86% vs. <1 year: 0.29%; *p* = 0.01), and region (Central: 3.36% vs. Eastern: 0.86%; *p* < 0.05). Notably, no association was found with breed or farming system (*p* ≥ 0.08), contrasting prior studies and suggesting region-specific transmission dynamics. Females exhibited heightened seropositivity, implicating prolonged herd retention and vertical transmission risks. Geographic disparities highlighted industrialized farming in central Algeria as a potential transmission amplifier. Strikingly, seronegative animals in high-prevalence herds hinted at genetic resistance, warranting further investigation. This study provides foundational insights into MVV epidemiology in North Africa, underscoring the need for targeted surveillance, ewe-focused control measures, and genetic research to mitigate transmission. The absence of prior national data elevates its significance, offering actionable frameworks for resource-limited settings and enriching the global understanding of SRLV heterogeneity.

## Simple Summary

This first national seroprevalence study of maedi-visna virus (MVV) in Algerian sheep herds demonstrates critical insights into a pathogen that causes chronic disease and economic losses globally. Through the analysis of 1400 sheep across four regions via indirect ELISA, we observed an overall seroprevalence of 9.07% (95% CI: 7.57–10.57), with significant disparities by sex (females: 20.44% vs. males: 3.68%; *p* < 0.05), age (peak in 1–5-year-olds: 10.43%; *p* = 0.019), and geography (Central region: 3.36% vs. East: 0.86%; *p* < 0.05). The females’ prolonged herd retention and vertical transmission risks, coupled with industrialized farming in high-prevalence regions, drive these trends. Notably, breed and farming systems showed no association (*p* ≥ 0.08), challenging existing paradigms and suggesting region-specific transmission dynamics. Strikingly, seronegative animals in high-prevalence herds hint at genetic resistance, a novel finding warranting exploration. This study fills a critical gap in North African SRLV epidemiology, emphasizing the need for ewe-centric control measures, targeted surveillance, and genetic research to curb transmission. The absence of prior national data elevates its significance, offering actionable strategies for resource-limited settings and advancing the global understanding of MVV heterogeneity. These findings underscore the urgency of context-specific interventions and provide a foundation for future studies on host–pathogen interactions and resistance mechanisms in endemic regions.

## 1. Introduction

The retroviridae family is divided into several subgroups including lentiviruses, where small ruminant lentiviruses (SRLVs) constitute one of the most heterogeneous groups of pathogens in this group [1]. Responsible for chronic inflammatory pathologies of insidious and progressive evolution [2], called visna/maedi (VM) in sheep and caprine arthritis-encephalitis (CAE) in goats, it is multisystemic and often affects several organs at the end of its evolution [3]. The hypothesis of interspecific transmission of lentiviruses from small ruminants has been recognized [4,5].

SRLVs are not transmitted to humans [6] because membrane receptors are absent in human cells [7]; their negative impact is mainly due to economic losses resulting from increased mortality rates of young animals, the low average daily weight gain of lambs as well as care costs [8]. Maedi-visna virus has been identified worldwide, except for Australia and New Zealand [9]. Most European countries have reported the presence of MVV in their sheep flocks (20 to 60%), but the seroprevalence of many regions is not available [10]. MVV has also been reported in African countries with a variable prevalence of 5 to 15% [8,11].

Maedi-visna virus is a non-congenic pathogen and does not induce immunosuppression. The main routes of transmission for animals are generally the oral route, through the ingestion of virus-infected colostrum or milk, or more particularly, through the respiratory tract from the inhalation of airborne viral particles [6]. Most maedi-visna virus infections are subclinical, and visible symptoms are more common in sheep older than 4 years [12]. The main symptoms reported are cough, bronchial exudate, depression, and fever, which usually only occur in the presence of an associated bacterial infection [13]. Indurative, non-inflammatory breast sequelae are generally observed in affected females [14]. Due to the individual progression of the disease, infected animals remain in the herd for some time, causing transmission of the disease within the herd until they succumb to the disease or are culled, thereby contributing to transmission of the virus in herds [6]. No treatment or vaccine has a proven effectiveness against the maedi-visna virus; the only control measures mainly target the management of infected farms and profiling measures for unaffected farms [15,16]. The harmful impact of MVV is often overlooked by producers and are therefore reluctant to diligently establish control measures; accordingly, specific control programs should be based only on a few easy-to-implement, low-effort measures to encourage adherence [13]. Some measures can be undertaken in flocks where a moderate seroprevalence of MVV is recorded, and targeted culling strategies aimed at only seropositive sheep have been proposed to gradually reduce the seroprevalence without increasing the culling rate [17].

## 2. Materials and Methods

### 2.1. Ethics Approval

All experiments in this study were carried out in accordance with the guidelines established by Directive 2010/63/EU of the European Parliament for the Animal Ethics Committee for the use of animal experimentation [17].

### 2.2. Sampling Design and Sample Size

The sample size was calculated using the formula for cross-sectional studies:

$$n=\frac{Z^{2}.p.(1-p)}{d^{2}}$$

where (Z = 1.96) (95% confidence level), *p* = 0.5 (expected prevalence, conservative estimate due to lack of prior national data), and *d* = 0.05 (precision). This yielded (*n* = 384) per region. To ensure robustness, we rounded to *n* = 400 for the East, Center, and West, and *n* = 200 for the South (due to lower sheep density).

We conducted a stratified, multistage cluster survey of sheep herds across different Algerian areas. The master sampling frame comprised more than 2000 sheep flocks registered with the Algerian Ministry of Agriculture, each assigned a unique herd ID and geocoordinates. Herds were stratified by administrative region (North, Central, South) and by flock-size category (<100, 100–300, >300 heads). Within each stratum, 10–15 herds were selected by probability-proportional-to-size (PPS) sampling, yielding 50 herds in total. Sample size calculations assumed an expected national seroprevalence of 10%, a desired precision of ±4%, 95% confidence, and a design effect of 1.5, which required N = 1400 animals. To ensure adequate power for region-specific estimates, we sampled 28 sheep per herd (total N = 1400). Within each selected herd, individual animals aged ≥one year were chosen by systematic random sampling (sampling interval = herd census size/15). Jugular blood was collected into plain tubes, labelled with the herd and animal IDs, transported on ice, and stored at −20 °C until ELISA testing.

**Figure 1:**
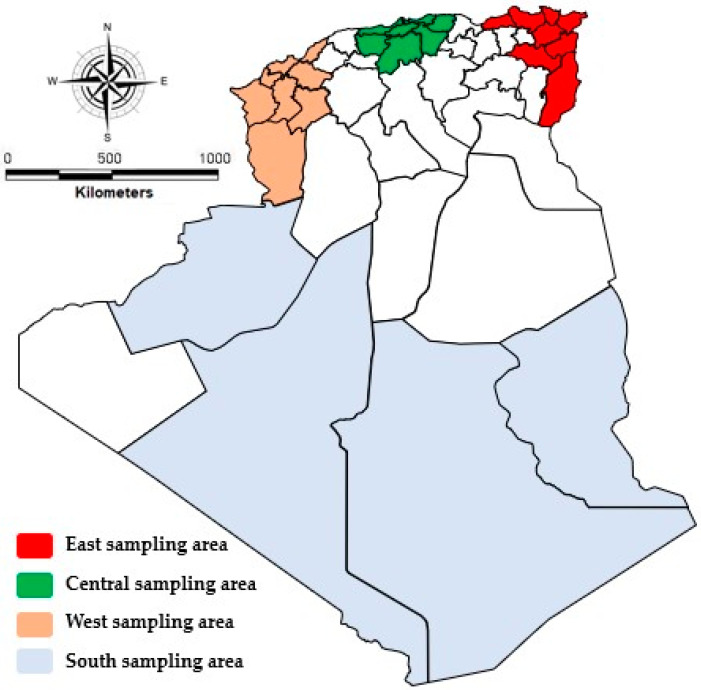
Repartition of sampled areas in Algeria.

A total of 1400 serum samples from sheep were taken in this study as follows: *n* = 400 from farms in the East, *n* = 400 sheep came from farms in the Center, *n* = 400 sheep from farms in the West, and *n* = 200 from farms in the South (Figure 1). The sheep were sampled according to age (1<, 1–5 years, and >5 years), sex (males *n* = 950 and females *n* = 450), origin of the animals (geographic region), and their breeds. While our study focused on major sheep-producing regions, future work should expand to underrepresented areas to refine the national seroprevalence estimates.

In total, *n* = 120 sheep on each farm were less than 1 year old, *n* = 920 sheep were between 1 and 5 years old, and *n* = 360 were over 5 years old; *n* = 950 males and *n* = 450 females. In addition, *n* = 632 of the sampled were of the Ouled-Djellal breed, *n* = 355 of the Hamra breed, *n* = 285 were of the Dmen breed, and *n* = 128 of the sheep were of the Serdi breed. Sheep were randomly selected from adult flocks (1 year old) to target sexually mature animals, which are at higher risk for MVV transmission.

### 2.3. Blood Collection and Serum Preparation

Keeping the animals upright and properly restrained by their owners, all blood samples were collected aseptically from the jugular vein. A disposable syringe was used to collect 5 mL of blood from the jugular vein of each animal. The syringes were held in a vertical position at 27 °C for approximately 2 h. The separated serum was quickly centrifuged at 1500× *g*, appropriately cold. Afterward, all of the collected serum was stored at −20 °C until ELISA testing [18].

### 2.4. Screening Test

The collected serum samples were analyzed to assess their content of MVV antibodies using an indirect screening ELISA (maedi-visna/CAEV) (IDvet, Grabels, France), with 96-well coated flat-bottom microplates. The test was based on the use of an immunogenic peptide from the transmembrane protein and the recombinant protein P28, which is part of the composition of the viral capsid. Test sera were diluted 1:20 with phosphate-buffered saline (PBS). A total of 200 µL of this dilution was placed in a coated ELISA microplate well. A 1:20 dilution series of 200 µL of positive and negative ovine reference antisera was placed in duplicate in each test plate. The plates were incubated at 37 °C for 1 h on a rotary shaker. After a subsequent wash, 100 μL of peroxide-labeled rabbit anti-ruminant immunoglobulin G, diluted 1:100 in PBS blocking reagent, was added to each well and incubated at 37 °C for 1 h on a rotary shaker. The reaction was revealed with 100 µL of tetramethylbenzidine per well after washing, and then stopped after 20 min with 100 µL of 0.5 M sulfuric acid per well. The optical density (OD) reading was carried out using a spectrophotometer at 450 nm.

The diagnostic performance of the ELISA was validated using sensitivity (100%) and specificity (97.8%) metrics provided by the manufacturer, derived from comparative testing against a recombinant GAG (group-specific antigens)–GST (glutathione S-transferase) fusion protein-based ELISA [18]. To account for potential false positives/negatives, apparent seroprevalence was adjusted to estimate the true seroprevalence using the Rogan and Gladen correction formula [19]:

$$True\;Prevalence=\frac{Apparent\;Prevalence+Specificity-1}{Sensitivity+Specificity-1}$$

Optical density (OD) values were normalized to calculate the sample-to-positive (S/P) ratio:

$$S/P(\%)=\left(\frac{ODsample-ODnegative\;control}{ODpositive\;control-ODnegative\;control}\right)\times 100$$

This standardized approach ensures reproducibility and minimizes inter-assay variability.

A value greater than or equal to 40% is considered a positive result [19], whereas if it is less than 30%, it is considered negative [20]. Samples with inhibition rates of 40–50% (equivocal) were retested in duplicate. Persistently equivocal results (2.1% of samples, n = 29) were excluded to avoid misclassification. A herd was considered positive when at least one animal in the herd was tested positive. This test was chosen as it is routinely used in serosurveys of MVV infection worldwide as well as its high sensitivity and specificity [21].

### 2.5. Risk Factors

In order to determine their association with MVV seroprevalence in sheep, the following variables were evaluated: breed, age, sex, nature of breeding (mixed or exclusive sheep), and breeding system (intensive, semi-intensive and extensive). Results became statistically significant when *p* ≤ 0.05 within 95% CI.

### 2.6. Statistical Analysis

The data obtained were analyzed using Microsoft Excel (Microsoft Corporation, Redmond, WA, USA). The risk factors for the seroprevalence of ovine lentivirus were assessed using SPSS version 15.0 (IBM SPSS, Armonk, NY, USA). Pearson’s chi-squared test was used to compare the serological status of MVV infection. *p* values less than 0.05 were considered statistically significant. Pearson’s chi-squared test was also applied to check for differences between the outcome variables in a univariate analysis. A probability of less than 0.05 was then considered statistically significant [18]. The relationship between the prevalence of MVV and different risk factors (region, sex, age, breeding methods) was determined by the chi-square test, and the strength of this relationship was determined by the Cramer’s V test where the Cramer’s V thresholds were as follows: <0.1 (negligible), 0.1–0.3 (weak), 0.3–0.5 (moderate), and >0.5 (strong). The strength of association was determined using Cramer’s V, with the thresholds defined as follows: <0.1 (negligible), 0.1–0.3 (weak), 0.3–0.5 (moderate), and >0.5 [22].

For regional pairwise comparisons, Bonferroni correction was applied to control for Type I errors (adjusted α = 0.0083).

## 3. Results

The existence of lentivirus antibodies was examined in the serum samples. The following variables were included in the data analysis at the individual sheep level: age, gender, breed, type of breeding, and study area (regions). A total of 950 males and 450 females from four different regions of Algeria—East (n = 400), Center (n = 400), West (n = 400), and South (n = 200)—were used to gather the samples. In terms of age, 120 sheep were younger than a year old, 920 animals were between one and five years old, and 360 sheep were older than five years.

### 3.1. Seroprevalence of Lentivirus in Algerian Sheep Herds

ELISA detected specific antibodies against the MVV antigens in 127 animals, giving an overall (raw) individual seroprevalence of 9.07% among sheep raised on farms in Algeria, as shown in Table 1.

### 3.2. Risk Factor Analysis

A statistically significant correlation was found between a number of the variables examined when the risk factors were analyzed using chi square at *p* < 0.05.

### 3.3. Variation of MVV Seroprevalence According to Sex of the Animal

Analysis of risk factors using chi-square at *p* < 0.05 showed that there was a statistically significant association between sex of the animal and the seroprevalence of MVV among sheep populations where 2.50% (35/1400) of the male sheep were seropositive for MVV while 6.57% (92/1400) in female sheep (Table 2).

#### 3.3.1. Regional Variation of Ovine Lentivirus Infection

Lentivirus MVV-positive animals were found in all study areas. For the eastern, central, western and southern regions, the seroprevalence proportions were 0.86% (12/1400), 03.21% (45/1400), 3.36% (47/1400), and 1.64% (23/1400). The statistical significance of these results was very high and attests to the geographical variation on the prevalence of MVV infection (Pearson chi-square test *p* < 0.05 and Cramer value = 13.39%) (Table 3).

Maedi-visna virus (MVV) seropositivity was detected across all sampled regions of Algeria, with significant geographical heterogeneity in prevalence. Seroprevalence proportions were quantified as follows:

- Eastern region: 3% (12/400);
- Central region: 11.25% (45/400);
- Western region: 11.75% (47/400);
- Southern region: 11.5% (23/200).

Statistical analysis confirmed robust regional variation (Pearson’s χ^2^ test: χ^2^ = 35.2, df = 3, *p* < 0.001; Cramer’s *V* = 0.134), indicating a small-to-moderate effect size. These findings underscore the influence of regional risk factors—potentially including husbandry practices, trade networks, or environmental conditions—on the MVV transmission dynamics.

Pairwise comparisons with Bonferroni correction confirmed that the Eastern region’s seroprevalence (3%) was significantly lower than that in the Central (11.75%, *p* < 0.001), West (11.25%, *p* < 0.001), and South (11.5%, *p* < 0.001). No significant differences were observed among the Central, West, and South (*p* > 0.70).

- Central vs. East: χ^2^ = 28.4, *p* < 0.001 (adjusted α = 0.0083);
- West vs. East: χ^2^ = 26.1, *p* < 0.001;
- South vs. East: χ^2^ = 24.7, *p* < 0.001;
- Central vs. South: χ^2^ = 0.12, *p* = 0.73.

These results confirm that the Eastern region’s seroprevalence (3%) was significantly lower than all other regions, while differences between Central, West, and South were nonsignificant.

#### 3.3.2. Variation in MVV Seroprevalence Depending on Sheep Breeds

The total of animals seropositive for maedi-visna virus infection and the prevalence by sheep breed studied (Ouled Djellal, Hamra, D’man, and the Serdi cross breed) is reported in Table 4, where these data showed diversity and variability in the results. The Serdi cross breed showed the lowest seropositive proportion of 1.07% (27/1400), the highest value was recorded in the Ouled Djella breed sheep of 3.43% (48/1400), while for the Hamra and D’man animal breeds, the seropositivity values were 2.64% (27/1400) and 1.93% (27/1400) respectively. The variability in results may reflect heterogeneous exposure risks; however, the differences did not reach statistical significance (*p* > 0.05), potentially due to the limited power to detect small effect sizes.

#### 3.3.3. Effects of Breeding Methods

Our study revealed a relatively high rate of overall seropositivity to infection in maedi-visna depending on the types of sheep farms in Algeria. To study the veracity of seropositivity between different types of livestock, the prevalence of MVV infection was analyzed, and we noted that sheep raised in extensive mode had a higher rate of infection by ovine lentivirus (5.79%, 81/1400) compared with sheep reared in semi-intensity (2.07%, 17/1400) or intensive (1.21%, 29/1400) systems, respectively, but these differences were not statistically significant (*p* = 0.08) (Table 5).

#### 3.3.4. Age Variation of Ovine MVV Infection

The study’s data showed a strong relationship between sheep age and the prevalence of maedi-visna virus (MVV) infection. The seroprevalence proportions varied by age group, with lambs under 1 year old having the lowest prevalence (3.33%), sheep aged 1 to 5 years having the greatest prevalence (10.43%), and sheep older than 5 years having the second-highest prevalence (7.50%). The chi-square test showed that this trend was statistically significant (*p* = 0.019), and the association’s strength was moderate (Cramer’s V = 7.53%). This finding is further supported by the odds ratio, which indicates that sheep between the ages of 1 and 5 had a 3.38-fold higher chance of testing positive for MVV than younger lambs (Table 6).

## 4. Discussion

Maedi-visna is a viral disease of sheep that is characterized by a lymphoproliferative pneumonia, meningeal arteritis with encephalitis, non-suppurative arthritis, and lymphocytic mastitis. However, due to the individual and slowly progressive nature of MVV infection due to delayed seroconversion, animals carrying the virus remain asymptomatic and may show no apparent clinical signs for a long period of up to several months or years [23] before seroconversion becomes evident or never occurs at all, which is a major feature of SRLV infections [23,24,25,26]. Animals may also fail to develop antibody levels determined by serological diagnostic tests [10,23].

This was the first epidemiological study of the degree of MVV infection in sheep herds in Algeria. The results of this study showed a relatively high seropositivity with influences of certain risk factors and the absence of the impact of others [4]. The higher MVV seroprevalence proportions reported in some countries is often associated with risk factors such as the type of farming, breed of animal, sex of animal, and age of animal [8,20]. This is consistent with the results of this study, in which CAEV seropositivity was observed more in local breed sheep (Ouled Djellal, 3.43% (48/1400)), sheep aged between 1 and 5 years, 6.86% (96/1400), and in females (6.57%, (92/1400)).

Recent studies in Africa have provided evidence of the existence of SRLV infections in Sudan [27], Kenya [28], Morocco [29], Nigeria [29], and Mozambique [29]. One of the common denominators in all of these countries is the fact that they imported animals from Europe, which would have facilitated the introduction and spread of the disease in local herds. This highlights the fact that the spread of SRLV initially occurred primarily through the importation of animals [29,30]. Animal importation was also implicated in the spread of infection in several previous studies that showed that in Japan [31] and Mexico [32], CAEV was found in imported goats for their breeding programs. Several risk factors, such as breed, animal sex, herd management, environmental conditions, or underlying genetic differences in infecting virus strains, may explain the significant differences in seroprevalence observed in our study [24,33].

The prevalence of individual seropositivity reported increased significantly with the age of the animals (0.29% for animals under one year and 6.86% for animals aged 1 and 5 years); this difference was statistically significant (*p* = 0.01). The increase in seroprevalence seen with age is likely due to the higher probability of horizontal infection with longer exposure times and increases in antibody production over time, rather than an increase in physiological age related susceptibility [34,35].

A significant increase (*p* < 0.05) was also observed with animal sex (2.5% of males and 6.57% of females) that can be explained by the fact that females are reared longer in herds unlike males, which are usually seduced at fattening and slaughter. The high incidence of MVV observed in females may be an important factor of vertical transmission and constitute very important sources of contamination.

From the results in Table 3, the MVV seroprevalence in Algerian sheep varied significantly (*p* < 0.005, Cramer’s V = 13.39%) between different geographical areas. The East region exhibited significantly lower seroprevalence (3%) compared with Central (11.75%), West (11.25%), and South (11.5%) (*p* < 0.001), while no significant differences were observed among the latter three regions (*p* > 0.70).This was probably due to different farming methods and socioeconomic backgrounds. As evidenced by European studies [36] showing that intensive practices increased lentiviral spread, Central Algeria’s industrialized farming systems, which are typified by high-density housing and frequent animal movement, may aid aerosol transmission [37]. The East and South, on the other hand, had lower seroprevalence, which is consistent with smallholder-dominated areas where fewer biosecurity measures are counterbalanced by lower animal densities and less inter-farm contact, thus possibly limiting the opportunities for transmission [20,21]. In these regions, socioeconomic limitations like limited access to centralized breeding programs or veterinary care could further cut off herds from exposure routes. These results highlight the need for region-specific control strategies: smallholder systems could put community education and decentralized surveillance first to strike a balance between resource constraints and efficient mitigation, while industrialized zones might benefit from improved biosecurity and ventilation protocols [37]. Our regional disparity (OR = 2.1, East vs. South) parallels findings in SRLV-infected herds in Greece, where trade hubs showed elevated transmission [36].

The proportional increase in seroprevalence with age up to five years can probably be explained by a longer period of exposure to horizontal transmission and the delay in seroconversion after infection, which makes the detection of antibodies very difficult and the elimination of affected animals impossible. Others have also reported an increase in seroprevalence with age [38].

The time between infection and the development of detectable levels of anti-MV antibodies can vary from months to years [39]. In flocks infected with MVV, the majority of infected sheep showed a serological reaction at 24 to 36 months of age [40]. Houwers et al. [41] found a statistically significant link between ewe and lamb in the transmission of MVV.

Small ruminant lentiviruses are easily transmitted through colostrum and milk [42,43] and has been isolated from sheep’s milk during the first five months of lactation [35]. A contact of just ten hours between an infected mother and her offspring has a 28.7% probability of cross-infection [43], which is a very important risk factor in our study given the significant impact of the sex of the animal on the total seroprevalence (*p* < 0.05, Cramer’s V = 27.25%).

Sheep of all ages can apparently be infected by the inhalation of droplets from the respiratory tract of infected individuals [44].Our results indicated a decrease in prevalence in sheep aged over five years compared with those aged between 1 and 5 years (7.50% and 10.43%, respectively). This reduction could be explained by the slaughter and death of sheep with MVV infection that had progressed to the clinical stage following infection earlier in their lives. According to the obtained data, there is a notable age-related pattern in the prevalence of maedi-visna virus (MVV) in sheep. Seropositivity was highest in sheep aged 1–5 years (10.43%), followed by those aged >5 years (7.50%), and lowest in lambs under 1 year (3.33%) (*p* = 0.019, Cramer’s V = 7.53%). Due to delayed seroconversion as MVV antibodies develop months to years after infection, and prolonged exposure to horizontal transmission (e.g., shared feeding places, respiratory/milk secretions), the odds ratio (3.38) showed that lambs aged 1 to 5 had the highest infection risk. Although vertical transmission (in utero/milk) is possible, the lower incidence in lambs is a result of decreased environmental exposure. The decrease in older sheep (>5 years) indicates that clinically afflicted animals should be culled due to pneumonia and wasting brought on by MVV. These results are consistent with earlier research that linked cumulative exposure to age [4]. In addition to direct losses due to the pneumonia associated with the disease and concomitant pathologies, a large number of emaciated and sick sheep are expected to retain sequelae of the infection and show significant economic losses to the farm [39].

Breeding is one of the risk factors identified in sheep infection with MVV. The reported infection rates in animals raised in an extensive and semi-intensive and intensive system differ due to different conditions and promiscuity as well as the risk of the direct transmission of viral particles [4,6]. In our study, the prevalence of seropositivity in animals raised in an extensive livestock system was higher (5.79%, 81/1400) compared with animals raised in an intensive livestock (2.07%, 29/1400) and semi-intensive system (1.21, 17/1400), respectively. This variability can be explained by the fact that females randomly breastfeed all newborn lambs, thus increasing the risk of vertical transmission and maintaining infection within the herds [21]. The non-significant breed trend aligns with SRLV studies in Italian goats, where management practices outweighed the genetic factors [45,46].

The discovery of seronegative animals within high-prevalence herds raises compelling questions about genetic resistance, a phenomenon also observed in Italian sheep breeds, where TMEM154 gene polymorphisms have been linked to reduced SRLV susceptibility [45]. This parallel underscores the universality of host genetic factors in modulating lentiviral outcomes and reinforces the need for breed-specific genomic studies in Algerian populations.

While not statistically significant (*p* = 0.08), the trend toward higher seroprevalence in extensive systems (5.79% vs. 1.21% intensive) aligns with studies linking prolonged lambing contact and colostrum sharing to transmission [5].

Several serological studies have shown variability in prevalence in some breeds compared with others. In our study, prevalence differed between breeds (Ouled Djellal, Hamra, D’man, and the breed crossed with Serdi with seroprevalence values of 3.43%, 2.64%, 1.93%, and 1.07, respectively) without statistical significance (*p* = 0.308, Cramer’s V = 5.10%) [21].

Globally, associations in our study were weak (V = 0.12–0.25), suggesting limited practical significance.

The differences in sensitivity between breeds may be related to the composition of the flocks and the fact that the farms are pure sheep breeds or often mixed with other breeds. Consequently, the proportions obtained for cross-breeds may have limited validity. The selection criteria for the breeds tested were based on the phenotypic appearance of the sheep and the origin of the flocks.

According to Cutlip et al. [47], all breeds of sheep are susceptible to MV infection, and the serological evidence of differences between breeds does not correlate with the susceptibility data because of the history of exposure to the virus. Houwers et al. [24] found a difference in seroprevalence between the Finnish Landrace and Ile de France breeds, although exposure was comparable. They concluded that the Ile-de-France breed was relatively susceptible to infection, which may be related to the influence of one or more recessive genes on the ovine lineage and virus strain characteristics. Some Icelandic sheep breeds are more resistant to the disease than others, and crosses between Icelandic sheep and Border Leicester breeds are particularly resistant. This resistance results in a slower onset of the disease. Cutlip et al. [47] showed experimentally that infected border sheep were more susceptible to disease symptoms and lesions than Colombian sheep. Following an analysis of MVV disease affecting native Icelandic sheep breeds, it was suggested that host genetics are a key factor in determining resistance and clinical manifestations of the disease [44]. Epidemiological data also suggest that resistance to MV infection is heritable [20].

The seroprevalence proportions in mixed farms (sheep and goats) was higher compared with the percentage of animals affected in pure sheep farms (7.93% and 1.14%, respectively), however, the difference reported was not statistically significant.

While our study identified significant univariate associations between sex, age, region, and MVV seroprevalence, the low overall prevalence limited our ability to robustly evaluate the interaction effects. Future longitudinal studies with larger sample sizes are warranted to explore synergistic risk factors, such as age–sex–region interactions, which may further refine targeted control strategies.

It is known that visna-maedi can infect goats and CAEV can infect sheep, although reported cases of natural transmission between species are rare [48,49,50], and recombination between visna-maedi and CAEV has recently been demonstrated [48]. In addition to the fact that CAEV has recently been reported in Japan [51,52], any eradication program should simultaneously target visna-maedi and CAEV infections, thereby eradicating any suspected SRLV outbreak.

## 5. Conclusions

This first study in Algeria assessed the prevalence of maedi-visna infection in sheep flocks, highlighting the effect of different risk factors on the spread of infection.

The results obtained in this study constitute the first observations of the infection of sheep herds with the maedi-visna virus in Algeria, and suggest relatively high proportions of seroprevalence of maedi-visna in sheep farms (regardless of whether they are pure or mixed with goats). MVV infection appears to affect several breeds of sheep, although large-scale epidemiological studies involving more breeds are needed to reliably estimate the spread of the virus in the country.

The higher incidence in ewes in our study indicates the need for more stringent preventive and eradication measures against horizontal transmission in infected farms due to the high risk of virus transmission to the newborn through the colostrum.

Our results confirm the need for continuous screening controls until the eradication of VM in infected herds because of the latency of the disease, and especially because of the very variable delays of seroconversion, many infected animals may not be detected, increasing the risk of latent transmission. Future studies should also correlate serostatus with clinical manifestations via longitudinal monitoring.

The discovery of seronegative animals within high-prevalence herds raises interesting questions about the potential for genetic resistance to MVV. This discovery is reinforced by studies on Icelandic and Border Leicester sheep breeds in which hereditary resistance to lentiviral disease has been documented [47,53]. For instance, Icelandic sheep are characterized by recessive alleles, having delayed onset of disease due to viral susceptibility, an effect that might reflect resilience in Algerian flocks. In order to illuminate such mechanisms, we recommend forthcoming genome-wide association studies (GWASs) for identifying resistance-associated alleles within native breeds such as Ouled Djellal or Hamra.

These results have the potential to revolutionize MVV control programs by incorporating genetic selection in breeding programs [37], selecting resistant lineages for reducing transmission bottlenecks. This approach, besides being in accordance with sustainable agriculture practices, is a cost-effective measure compared with resource-exhausting eradication strategies in endemic regions. Our findings emphasize the need to explore host–pathogen genetic interactions to implement targeted interventions in North African sheep populations.

Based on the results obtained in this study, it is recommended that more extensive testing for antibodies to MVV be carried out in each region of Algeria in order to determine the true seroprevalence of MVV in the country. In addition, and most importantly, it is imperative that MVV genotyping be carried out on larger samples in order to identify the strain of MVV prevalent in Algeria. Seronegative animals in high-prevalence herds suggest potential genetic or immunological resistance, warranting genomic studies to identify protective alleles.

This study underscores the urgency of ewe-centric control measures (e.g., segregated lambing, colostrum pasteurization) and targeted surveillance in high-prevalence regions. The potential for genetic resistance in seronegative animals highlights a novel avenue for breeding programs. Future work should prioritize MVV genotyping and longitudinal cohorts to elucidate the transmission dynamics.

## Figures and Tables

**Table 1:** Total seroprevalence of the MVV among sheep in Algeria.

Variable	No. Tested	No. Positive	Prevalence (%)	95% CI
Total	1400	127	9.07	7.57–10.57

**Table 2:** Seroprevalence of MVV based on animal sex.

Variable		No. Tested	Prevalence (%)	95% CI	Cramer’s V (%)	Prevalence (%)	Odds Ratio
Sex	Male	950	3.68	2.49–4.88	27.25%	2.50	6.72
	Female	450	20.44	16.72–24.17		6.57	

**Table 3:** Seroprevalence of MVV infection based on the geographical region variable.

Variable		No. Tested	No. Positive	95%CI	V Cramer (%)	Regional Prevalence (%)	Global Prevalence (%)	Odds Ratio
Region	East	400	12	1.33–4.67	13.39%	3	0.86	4.31
	West	400	45	8.15–14.35		11.25	3.21	
	Center	400	47	8.59–14.91		11.75	3.36	
	South	200	23	7.08–15.92		11.5	1.64	

**Table 4:** MVV seroprevalence according to sheep breeds.

Variable		No. Tested	No. Positive	95% CI	χ^2^-*p*	Cramer’s V (%)	Prevalence (%)	Odds Ratio
Breed	Ouled Djellal	632	48	5.53–9.66	0.308	5.10%	7.59	1.62
	Hamra	355	37	7.24–13.6			10.42	
	D’man	285	27	6.07–12.87			9.47	
	Crossed Serdi	128	15	6.15–17.29			11.71	

**Table 5:** Seroprevalence of maedi-visna virus among sheep according to breeding methods.

Variable		No. Tested	No. Positive	Prevalence (%)	95 % IC	χ^2^-*p*	Cramer’s V (%)	Odds Ratio
Breeding method	Extensive	1009	81	08.03	6.35–09.71	0.087	5.90	1.58
	Semi-intensive	152	17	11.18	6.17–16.19			
	Intensive	239	29	12.13	7.99–16.27			

**Table 6:** Seroprevalence of MVV infection based on age variable.

Variable		No. Tested	No. Positive	Prevalence (%)	95% IC	χ^2^-*p*	Cramer’s V (%)	Odds Ratio
Age	<1	120	4	3.33	0.12–6.55	0.019	7.53	3.38
	1 to 5	920	96	10.43	8.46–12.41			
	>5	360	27	7.50	4.78–10.22			

## Data Availability

The data presented in this study are available within the article. Raw data supporting this study are available from the corresponding author upon reasonable request.

## Abbreviations

The following abbreviations are used in this manuscript:

SRLV	Small ruminant lentivirus
MVV	Maedi-visna virus
CAEV	Caprine arthritis encephalitis virus
